# The impact of postpartum smoking on breastfeeding initiation and duration: racial and ethnic analysis using PRAMS data in a nationally representative sample

**DOI:** 10.1186/s13006-026-00871-4

**Published:** 2026-07-15

**Authors:** Stefanie Gillson, Deanna Nardella, Maureen Canavan, Ariadna Forray

**Affiliations:** 1 Child Study Center, Yale School of Medicine, 230 South Frontage Road, New Haven, CT 06510 USA; 2 Department of Pediatrics, Yale School of Medicine, 100 College Street South, New Haven, CT 06510 USA; 3 Department of General Internal Medicine, Cancer Outcomes Public Policy and Effectiveness Research (COPPER) Center, Yale School of Medicine, 333 Cedar Street, New Haven, Connecticut, CT 06510 USA; 4 Department of Psychiatry, Yale School of Medicine, 333 Cedar Street, New Haven, CT 06510 USA

**Keywords:** Postpartum smoking, Breastfeeding initiation, Breastfeeding cessation, Race/ethnicity disparities, PRAMS, Maternal and child health

## Abstract

**Objectives:**

To examine the associations between postpartum smoking and breastfeeding initiation and duration by race/ethnicity among U.S. women.

**Methods:**

Cross-sectional survey weighted data from the 2016–2022 Pregnancy Risk Assessment Monitoring System (PRAMS) were analyzed. Breastfeeding initiation and duration were defined by self-report of ever breastfeeding and breastfeeding duration (weeks), postpartum smoking was any cigarette use in the prior three months. Multivariable logistic regression estimated the adjusted odds of breastfeeding initiation by smoking status. Cox proportional hazards regression quantified the median time to and adjusted hazard of breastfeeding cessation by smoking status. Significant interaction between smoking and race/ethnicity led to stratified analyses.

**Results:**

Among 203,802 women, 87.9% initiated breastfeeding; 11.0% reported postpartum smoking with highest among American Indian/Alaska Native (AI/AN) women (28.8%) and lowest among Asian women (1.3%). Smoking was associated lower odds of initiation across all groups except Native Hawaiian/Pacific Islanders (NH/PI), with the strongest association among Hispanic women (aOR 0.39; 95% CI, 0.33–0.45) and Asian women (aOR 0.40; 0.25–0.63). In Cox models, smoking was associated with higher hazards of breastfeeding cessation for all groups except NH/PI; the greatest hazards were seen in non-Hispanic White women (aHR 1.90; 95% CI, 1.81–2.00), and AI/AN women (HR 1.82; 1.55–2.13). Median time to cessation was shorter for smokers.

**Conclusions for practice:**

Postpartum smoking was associated with lower breastfeeding initiation and duration, with persistent racial/ethnic disparities. Culturally tailored interventions integrating smoking cessation support and breastfeeding promotion—including harm reduction strategies for continuing smokers—are essential to advance maternal and infant health equity.

## Significance

This study leverages nationally representative PRAMS data (2016–2022) to provide race-stratified analysis of how postpartum smoking influences both breastfeeding initiation and duration across racial and ethnic groups in the U.S.—including underrepresented American Indian/Alaska Native populations. By identifying differential smoking-related risks for early cessation and low initiation rates, the findings reveal persistent inequities that standard population-wide interventions may overlook. These insights underscore the need for culturally tailored cessation and lactation support, and inform policy makers and clinicians designing targeted strategies to improve breastfeeding outcomes and reduce infant and maternal health disparities.

## Introduction

Breastfeeding is widely recognized as a critical practice for promoting health and well-being for both infants and mothers. While most families in the United States (U.S.) initiate breastfeeding (83%), many do not sustain breastfeeding for the recommended six months as endorsed by leading health organizations such as the World Health Organization and the American Academy of Pediatrics [1, 2]. Longer durations of breastfeeding reduce a mother’s risk of diabetes, hypertension, breast and ovarian cancer, and reduce an infant’s risk of sudden infant death syndrome (SIDS), asthma, and several respiratory and gastrointestinal infections [3]. Yet women from socially and economically marginalized racial and ethnic groups breastfeed for shorter durations, are less often meet their infant feeding goals and therefore less likely to experience these health benefits [4, 5].

Postpartum cigarette smoking is a well-documented factor that can decrease breastfeeding initiation and duration [6]. Smoking during pregnancy is associated with lower breastfeeding initiation and shorter breastfeeding duration [7, 8], even among women who intend to breastfeed, with both physiologic and psychosocial mechanisms likely contributing. Both cigarette smoking and not breastfeeding significantly increase the risk of SIDS, which disproportionately impacts minority populations in the U.S., [9]. Tobacco companies have historically used marketing strategies aimed at specific racial and ethnic groups including Hispanic [10], Black [11], and American Indian/Alaska Native (AI/AN) [12] populations as well as women more broadly [13]. These targeted efforts contribute to the disparities in smoking rates and health outcomes and could influence the known breastfeeding disparities. Among U.S. women in 2021, 5.4% of women with a recent live birth reported smoking during pregnancy and 7.2% in the postpartum period [14]. AI/AN women reported the highest smoking rates during pregnancy (16.7%), while Asian (0.6%) and Hispanic (1.8%) women had the lowest rates [15]. Although many women quit smoking during pregnancy, relapse during the postpartum period remains common [16, 17].

While prior research has examined the relationship between smoking and breastfeeding, only a handful of studies have looked at how the association differs across racial and ethnic groups in the U.S. [18, 19], and even fewer have included populations such as AI/AN women [6]. Given persistent, targeted tobacco marketing strategies and a higher burden of systemic barriers to breastfeeding, it is critical to understand the relationship between postpartum smoking and breastfeeding outcomes and how it varies across racial and ethnic groups. Such knowledge could inform innovative strategies tailored to support smoking cessation and breastfeeding duration among the highest risk groups. This study aims to quantify the association between smoking exposure and breastfeeding initiation and duration among a nationally representative cohort of U.S. women using PRAMS data, and to examine how this relationship is modified by women’s race and ethnicity.

## Methods

### Study design and data source

We conducted a serial cross-sectional study of stratified, weighted data from the Centers for Disease Control and Prevention’s (CDC) PRAMS, which employs a sampling framework based on state-issued birth certificates to identify women 2 to 6 months postpartum across the U.S. The survey collects data on experiences across the pre-pregnancy, prenatal, and postpartum periods, in addition to information on obstetric history, infant health and development, substance use, and maternal stress. Each participating jurisdiction samples between 1,000 and 3,000 women annually, oversampling individuals from smaller sociodemographic populations, including racial and ethnic minorities, and assigns sample weights to responses to ensure representativeness of the jurisdiction’s demographics. Further methodological details can be found in previous literature [20].

### Study sample

This study included data from the 41 states participating in the CDC’s Automated Research File (ARF) and obtained additional PRAMS data from Oklahoma, given the state’s substantial AI/AN population, which aligns with the study’s focus on racial and ethnic disparities. PRAMS data from 2016 to 2022 was used, Oklahoma PRAMS dataset did not include 2019 and 2020 due to not meeting response threshold of 50%. The sample was limited to women who responded to the question about breastfeeding initiation, breastfeeding duration, and postpartum smoking. PRAMS refers to respondents as “women,” as it does not collect gender identity beyond this term. This study received an exemption from the Yale University Institutional Review Board in November 2024.

### Study measures

The two primary outcomes of this study were breastfeeding initiation and breastfeeding duration. Initiation was assessed using the PRAMS dichotomous question, “Did you ever breastfeed or pump breast milk to feed your new baby, even for a short period of time?” Participants who answered “yes” were classified as having initiated breastfeeding during the postpartum period. Duration was measured as the number of weeks a mother reported breastfeeding. Mothers who reported breastfeeding for less than one week were assigned a value of 0.5 weeks, and those still breastfeeding at the time of the survey were assigned their infant’s age in weeks at survey completion. The maximum reported breastfeeding duration was 40 weeks.

The primary independent variable was any cigarette use in the postpartum period, assessed through the question: “Have you smoked any cigarettes in the past three months?” Participants who answered “Yes” were classified as smokers in the postpartum period, while participants who responded as “No” to either this question or to “Have you ever smoked cigarettes?” were classified as non-smokers.

Several covariates were included in the analyses based on prior breastfeeding research [2, 18, 21–23]. Maternal sociodemographic characteristics were derived from birth certificate data. A combined race and ethnicity variable was created in accordance with federal guidelines for standardized reporting of race and ethnicity [22], including the following categories: Asian, Native Hawaiian and Pacific Islander (NH/PI), Hispanic, AI/AN, non-Hispanic Black, non-Hispanic White, and Other (non-Hispanic multiracial). Race/ethnicity categories were constructed as mutually exclusive groups, with Hispanic ethnicity categorized separately regardless of race. Individuals missing race and ethnicity data were excluded from analyses. Race and ethnicity information was collected from birth certificate data. PRAMS provided a single variable for other and mixed race, which could not be stratified further. Maternal age was categorized into six age groups (< 19 years, 20–24 years, 25–29 years, 30–34 years, 35–39 years, and ≥ 40 years). Educational attainment was categorized as less than high school diploma, high school graduate, some college, and college degree and above. Marital status was dichotomized as married versus unmarried. Marital status was dichotomized as married versus unmarried. Insurance type was divided into six categories (no insurance, Medicaid, private insurance, government insurance, Indian Health Service, and other/unknown). Body mass index (BMI) was categorized into four categories (underweight (< 18.5), normal (18.5–24.9), overweight (25.0–29.9), or obese (≥ 30.0). Participants were dichotomized based on whether they received services through Women, Infants and Children (WIC) program. Given high collinearity between income categories and participation in the WIC and higher percentage of missing observations in income, income was not included in this analysis. Maternal health covariates were all binary (yes/no) and included vaginal birth, planned pregnancy, if they had a previous live birth, discussed breastfeeding with a healthcare provider, any alcohol use during the three months before pregnancy, and access to prenatal care.

### Statistical analysis

The distribution of each categorical covariate by smoking status was described using chi-square tests to assess statistical significance. Multivariable logistic regression estimated the odds of breastfeeding initiation according to postpartum smoking status. Based on the hypothesized a priori interaction between smoking status and race/ethnicity, an interaction term was included in the model. Following identification of significant effect modification, stratified multivariable models were used to characterize these relationships.

Adjusted survival curves and median time to breastfeeding cessation by postpartum smoking status were generated and stratified by race/ethnicity. Race and ethnicity were identified as significant modifiers of the relationship between smoking status and time to breastfeeding cessation. Time to breastfeeding cessation was analyzed using multivariable Cox proportional-hazards regression, and the proportional-hazards assumption was assessed for all covariates. Statistical significance was defined as a two-sided test point of *P* < 0.05. Complete-case analysis was conducted under the assumption of missing-at-random, as all covariates had < 5% missing data. All analyses were conducted in STATA SE 18.0, incorporating PRAMS sampling weights and complex survey design.

## Results

The weighted sample included 203,802 women, of whom 87.9% reported breastfeeding and 11.0% reported postpartum smoking (Table 1). Nearly half of the respondents were non-Hispanic White (49.5%), followed by non-Hispanic Black (17.2%) and Hispanic (16.9%) populations. Asians accounted for 6.5% of the sample, while Other individuals accounted for 5.6%. Smaller proportions were AI/AN (3.9%) and NH/PI (0.4%). Cigarette use in the past three months varied significantly across racial and ethnic groups (*p* < 0.001), with the highest prevalence among AI/AN mothers (28.8%) and Asian mothers (1.3%) having the lowest. Additional information and significant differences in the distribution of covariates can be seen in Table 1.

**Table 1:** Sociodemographic and maternal characteristics of U.S. postpartum women from 2016 to 2022

Race/Ethnicity		Non- Hispanic White	Non-Hispanic Black	Hispanic	AI/AN	Asian	HI/PI	Other	*P* value
*N*	203,802	100,807 (49.5%)	35,124 (17.2%)	34,420 (16.9%)	7,908 (3.9%)	13,259 (6.5%)	906 (0.4%)	11,378 (5.6%)	
**Breastfeeding**									
No No	24,563 (12.1%)	10,841 (10.8%)	7,595 (21.6%)	2,869 (8.3%)	1,388 (17.6%)	775 (5.8%)	99 (10.9%)	996 (8.8%)	
Yes Yes	179,239 (87.9%)	89,966 (89.2%)	27,529 (78.4%)	31,551 (91.7%)	6,520 (82.4%)	12,484 (94.2%)	807 (89.1%)	10,382 (91.2%)	
**Postpartum Smoking**									<0.001
No	181,427 (89.0%)	88,502 (87.8%)	30,794 (87.7%)	32,871 (95.5%)	5,632 (71.2%)	13,083 (98.7%)	847 (93.5%)	9,698 (85.2%)	
Yes	22,375 (11.0%)	12,305 (12.2%)	4,330 (12.3%)	1,549 (4.5%)	2,276 (28.8%)	176 (1.3%)	59 (6.5%)	1,680 (14.8%)	
**Age**									
< 19yrs	9,047 (4.4%)	3,000 (3.0%)	2,265 (6.4%)	2,273 (6.6%)	755 (9.5%)	80 (0.6%)	39 (4.3%)	635 (5.6%)	<0.001
20–24	36,624 (18.0%)	15,248 (15.1%)	7,927 (22.6%)	7,952 (23.1%)	2,093 (26.5%)	765 (5.8%)	203 (22.4%)	2,436 (21.4%)	
25–29	58,492 (28.7%)	29,249 (29.0%)	10,426 (29.7%)	9,718 (28.2%)	2,353 (29.8%)	3,192 (24.1%)	269 (29.7%)	3,285 (28.9%)	
30–34	60,879 (29.9%)	33,604 (33.3%)	8,506 (24.2%)	8,354 (24.3%)	1,731 (21.9%)	5,401 (40.7%)	254 (28.0%)	3,029 (26.6%)	
35–39	31,711 (15.6%)	16,479 (16.3%)	4,706 (13.4%)	4,854 (14.1%)	806 (10.2%)	3,131 (23.6%)	112 (12.4%)	1,623 (14.3%)	
40+	7,041 (3.5%)	3,223 (3.2%)	1,293 (3.7%)	1,267 (3.7%)	170 (2.1%)	690 (5.2%)	29 (3.2%)	3,690 (3.2%)	
**Education**									< 0.001
Less than high school diploma	23,460 (11.6%)	6,169 (6.1%)	4,238 (12.2%)	8,923 (26.2%)	1,861 (23.7%)	809 (6.1%)	201 (22.2%)	1,259 (11.1%)	
HS graduate	48,665 (24.0%)	19,315 (19.3%)	12,104 (34.7%)	10,006 (29.4%)	2,776 (35.4%)	1,375 (10.4%)	294 (32.5%)	2,795 (24.7%)	
Some college	57,644 (28.5%)	27,862 (27.8%)	12,031 (34.5%)	8,961 (26.4%)	2,585 (32.9%)	2,026 (15.3%)	324 (35.8%)	3,855 (34.1%)	
College or more	72,671 (35.9%)	46,990 (46.8%)	6,460 (18.5%)	6,115 (18.0%)	630 (8.0%)	8,993 (68.1%)	86 (9.5%)	3,397 (30.0%)	
**WIC**									
No	128,076 (63.7%)	77,674 (77.8%)	15,042 (43.5%)	14,465 (42.7%)	3,199 (40.9%)	10,333 (79.8%)	497 (56.2%)	6,866 (61.5%)	< 0.001
Yes	73,013 (36.3%)	22,129 (22.2%)	19,516 (56.5%)	19,437 (57.3%)	4,620 (59.1%)	2,619 (20.2%)	388 (43.8%)	4,304 (38.5%)	
**Married**									
No	81,447 (40.0%)	26,197 (26.0%)	24,147 (68.8%)	17,989 (52.3%)	5,789 (73.3%)	1,469 (11.1%)	487 (53.8%)	5,369 (47.2%)	< 0.001
Yes	122,219 (60.0%)	74,538 (74.0%)	10,951 (31.2%)	16,414 (47.7%)	2,106 (26.7%)	11,790 (88.9%)	418 (46.2%)	6,002 (52.8%)	
**Insurance during pregnancy**									
No Insurance	3,689 (1.9%)	1,228 (1.2%)	330 (1.0%)	1,780 (5.5%)	81 (1.1%)	130 (1.1%)	24 (2.9%)	116 (1.1%)	< 0.001
Medicaid	86,173 (44.0%)	30,466 (31.0%)	22,918 (68.6%)	18,390 (56.7%)	5,646 (75.2%)	2,826 (22.8%)	510 (62.0%)	5,417 (49.8%)	
Private	94,406 (48.2%)	61,670 (62.7%)	8,623 (25.8%)	9,341 (28.8%)	1,166 (15.5%)	8,801 (71.1%)	233 (28.3%)	4,572 (42.0%)	
Gov Insurance	6,074 (3.1%)	3,093 (3.1%)	708 (2.1%)	1,436 (4.4%)	90 (1.2%)	305 (2.5%)	25 (3.0%)	417 (3.8%)	
Indian Health Service	649 (0.3%)	21 (0.0%)	2 (0.0%)	20 (0.1%)	458 (6.1%)			148 (1.4%)	
Other	4,752 (2.4%)	1,846 (1.9%)	823 (2.5%)	1,460 (4.5%)	68 (0.9%)	317 (2.6%)	30 (3.6%)	208 (1.9%)	
**BMI**									
normal	85,357 (43.8%)	47,528 (48.0%)	11,069 (33.1%)	11,545 (38.1%)	2,571 (33.8%)	7,851 (61.9%)	244 (29.6%)	4,549 (41.2%)	< 0.001
underweight	6,749 (3.5%)	3,233 (3.3%)	1,074 (3.2%)	1,007 (3.3%)	159 (2.1%)	882 (7.0%)	16 (1.9%)	378 (3.4%)	
overweight	49,880 (25.6%)	24,347 (24.6%)	8,761 (26.2%)	8,885 (29.3%)	2,063 (27.1%)	2,749 (21.7%)	211 (25.6%)	2,864 (25.9%)	
obese	52,945 (27.2%)	23,937 (24.2%)	12,542 (37.5%)	8,852 (29.2%)	2,811 (37.0%)	1,200 (9.5%)	353 (42.8%)	3,250 (29.4%)	
**Vaginal Delivery**									
No	72,041 (35.4%)	34,299 (34.0%)	14,243 (40.6%)	12,445 (36.2%)	2,247 (28.5%)	4,799 (36.2%)	358 (39.5%)	3,650 (32.1%)	< 0.001
Yes	131,620 (64.6%)	66,449 (66.0%)	20,861 (59.4%)	21,946 (63.8%)	5,650 (71.5%)	8,451 (63.8%)	548 (60.5%)	7,715 (67.9%)	
**Previous Live Birth**									
No	79,906 (39.3%)	41,526 (41.3%)	12,561 (35.8%)	12,328 (35.9%)	2,298 (29.2%)	6,307 (47.7%)	281 (31.1%)	4,605 (40.6%)	< 0.001
Yes	123,529 (60.7%)	59,127 (58.7%)	22,511 (64.2%)	22,011 (64.1%)	5,585 (70.8%)	6,924 (52.3%)	622 (68.9%)	6,749 (59.4%)	
**Received BF information**									
No	106,871 (52.8%)	56,217 (56.1%)	17,216 (49.4%)	17,342 (50.8%)	3,856 (49.2%)	6,185 (47.1%)	366 (40.8%)	5,689 (50.3%)	< 0.001
Yes	95,513 (47.2%)	44,021 (43.9%)	17,612 (50.6%)	16,801 (49.2%)	3,976 (50.8%)	6,951 (52.9%)	531 (59.2%)	5,621 (49.7%)	
**Alcohol use last 3 months**									
No	89,354 (44.2%)	33,078 (33.0%)	18,747 (53.9%)	19,553 (57.3%)	4,080 (52.1%)	8,675 (65.9%)	601 (67.2%)	4,620 (41.0%)	< 0.001
Yes	112,946 (55.8%)	67,155 (67.0%)	16,055 (46.1%)	14,553 (42.7%)	3,754 (47.9%)	4,488 (34.1%)	294 (32.8%)	6,647 (59.0%)	
**Tried to get pregnant**									
No	45,687 (22.8%)	17,846 (17.9%)	12,257 (35.5%)	7,195 (21.3%)	3,085 (39.8%)	1,991 (15.2%)	305 (34.6%)	3,008 (26.9%)	< 0.0001
Yes	154,973 (77.2%)	81,594 (82.1%)	22,252 (64.5%)	26,617 (78.7%)	4,659 (60.2%)	11,102 (84.8%)	577 (65.4%)	8,172 (73.1%)	
**Received Prenatal Care**									
No	33,571 (17.5%)	12,152 (12.7%)	7,799 (24.0%)	7,198 (22.1%)	2,302 (31.6%)	1,838 (14.6%)	330 (40.3%)	1,952 (18.4%)	< 0.001
Yes	158,601 (82.5%)	83,573 (87.3%)	24,756 (76.0%)	25,362 (77.9%)	4,987 (68.4%)	10,759 (85.4%)	489 (59.7%)	8,675 (81.6%)	
**Rural location**									
No	152,948 (79.2%)	72,690 (74.3%)	31,413 (89.5%)	24,935 (85.0%)	2,638 (40.5%)	12,400 (93.8%)	742 (82.5%)	8,130 (78.5%)	< 0.001
Yes	40,247 (20.8%)	25,102 (25.7%)	3,678 (10.5%)	4,391 (15.0%)	3,868 (59.5%)	823 (6.2%)	157 (17.5%)	2,228 (21.5%)	

### Associations between breastfeeding initiation and smoking

**Fig. 1:**
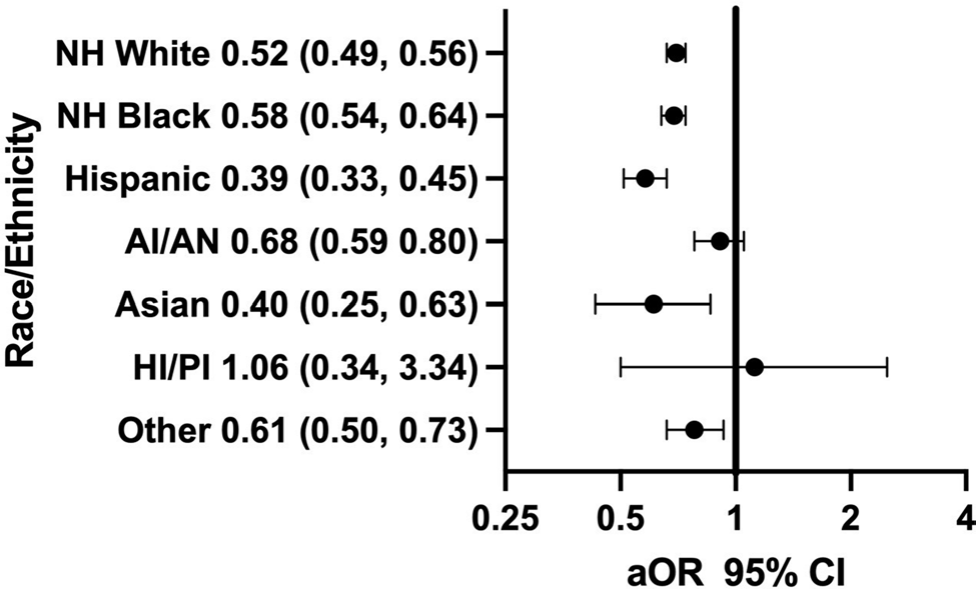
Adjusted odd ratios for association between smoking and breastfeeding initiation stratified by race/ethnicity among U.S. postpartum women from 2016 to 2022

Postpartum smoking was associated with significantly lower odds of breastfeeding initiation compared with non-smokers across all racial and ethnic groups, except among NH/PI (Fig. 1). Hispanic smokers had 61% lower odds of breastfeeding initiation compared with Hispanic non-smokers (adjusted odds ratio [aOR] = 0.39; 95% CI: 0.33–0.45), while Asian smokers had 60% lower odds of breastfeeding initiation compared with Asian non-smokers (aOR = 0.40; 95% CI: 0.25–0.63).

### The association of breastfeeding cessation and postpartum smoking

**Fig. 2:**
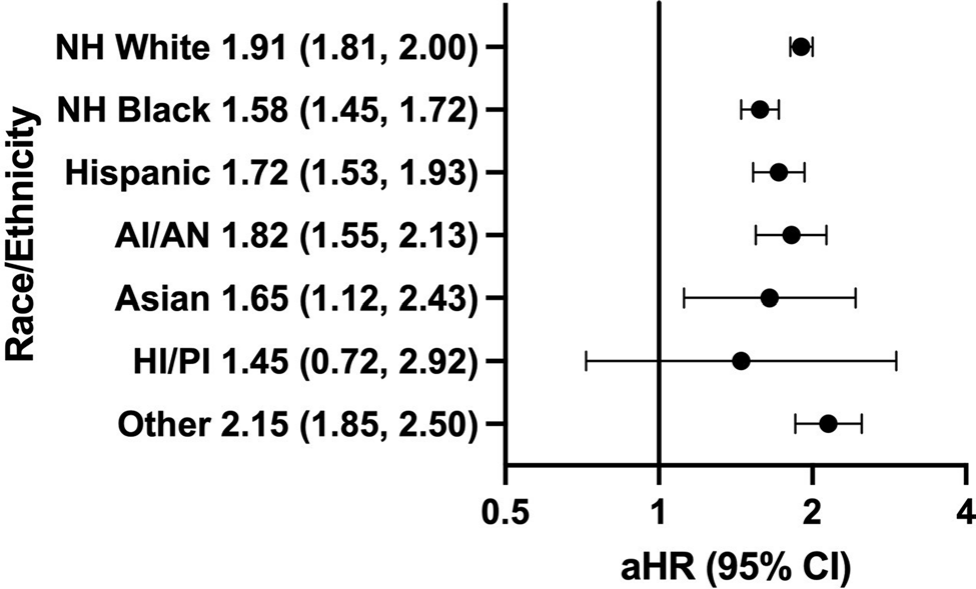
Adjusted hazard ratios for association between smoking and breastfeeding cessation, stratified by race/ethnicity among U.S. postpartum women from 2016 to 2022

**Fig. 3:**
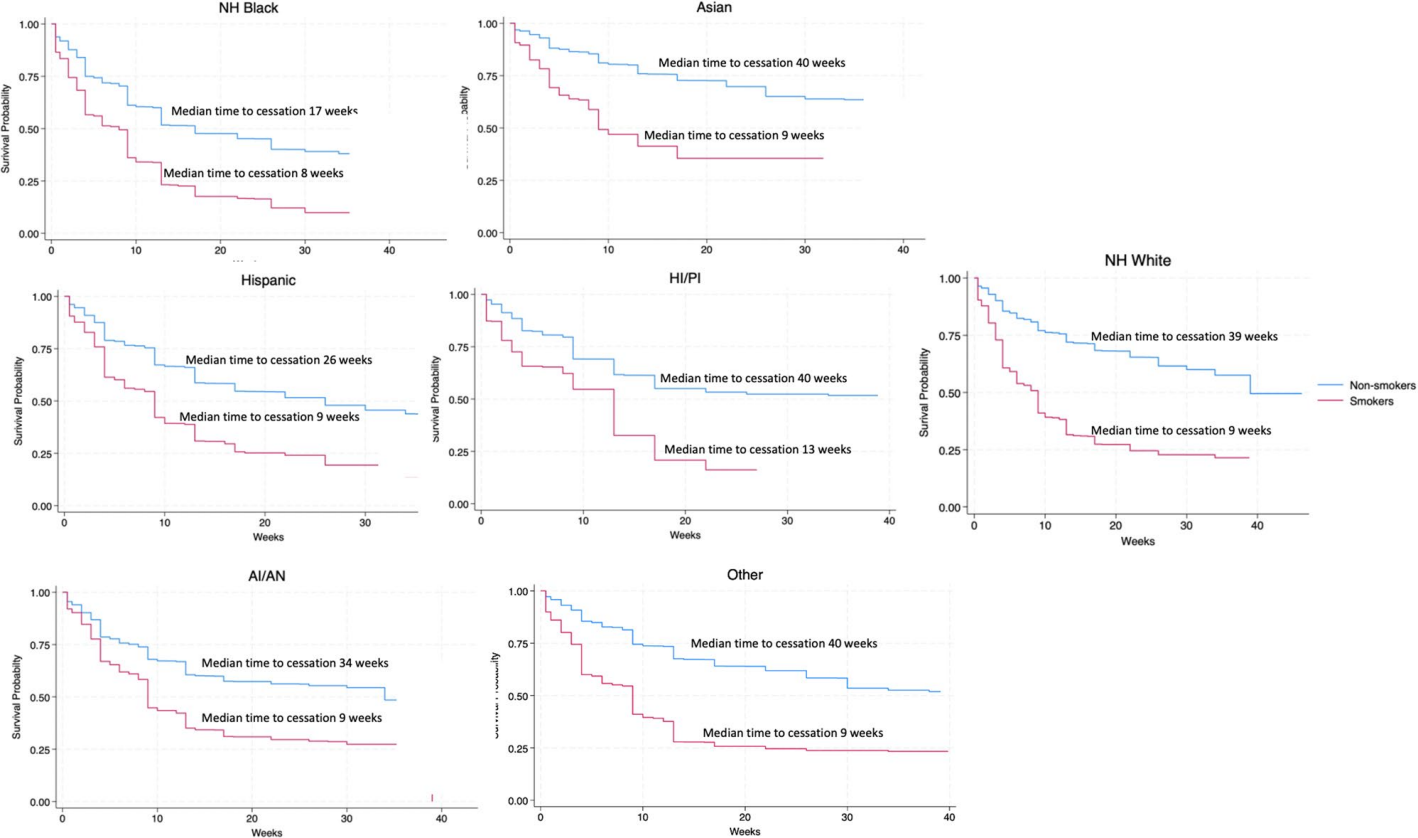
Kaplan- meier survival estimates for breastfeeding duration by smoking status and race/ethnicity among U.S. postpartum women from 2016 to 2022

Multivariable Cox Proportional Hazards models showed postpartum smoking was significantly associated with a higher risk of breastfeeding cessation for all racial and ethnic groups, except NH/PI (Fig. 2). Hazard ratios (HR) were highest among Other participants (aHR = 2.15, 95% CI: 1.85–2.50), followed by non-Hispanic White (aHR = 1.90, 95% CI: 1.81-2.00), and AI/AN (aHR = 1.82, 95% CI: 1.55–2.13) mothers. Adjusted Kaplan-Meier survival curve by smoking status identified differences in time to breastfeeding cessation across all race/ ethnicity groups with the median time to breastfeeding cessation consistently shorter for smokers across all racial/ethnic groups (Fig. 3).

## Discussion

This study highlights postpartum smoking as an important modifiable risk factor that is negatively associated with breastfeeding initiation and duration. The strongest negative association between any smoking in the postpartum period and breastfeeding initiation was observed among U.S. Hispanic and Asian mothers. Across all racial and ethnic groups, mothers who smoked had shorter breastfeeding durations, with the greatest risk of early cessation seen among Other, non-Hispanic White, and AI/AN mothers. Additionally, postpartum smoking prevalence is disproportionately high among AI/AN and non-Hispanic Black mothers. These findings reinforce the need for targeted public health strategies to improve maternal and infant health.

While all racial and ethnic groups in this study experienced significant effects of smoking on breastfeeding, AI/AN and non-Hispanic Black mothers had both higher smoking rates and shorter breastfeeding durations, reflecting patterns of systemic inequities. In the sample, AI/AN mothers had the lowest rates of prenatal care, higher rates of rural residency, and the greatest WIC participation, further highlighting barriers rooted in access and structural disadvantage. Non-Hispanic Black mothers continue to face reduced access to breastfeeding education and work accommodations [24]. Moreover, adjusted survival curves show that AI/AN and non-Hispanic Black mothers experienced the steepest early declines in breastfeeding, dropping off more sharply in the first weeks postpartum than other groups, highlighting the need for front-loaded, intensive support initiatives in these communities. In contrast, Hispanic and Asian mothers reported lower smoking rates and longer breastfeeding durations, potentially due to cultural norms, though barriers remain. Notably, however, smoking conferred the greatest relative risk of early breastfeeding cessation among these same Hispanic and Asian women, a paradox may reflect greater stigma toward smokers in these communities such as public breastfeeding among Hispanic mothers [25], gaps in culturally tailored cessation and lactation support for the few who do smoke, or that these smokers represent an especially vulnerable subset. These findings underscore the need for culturally tailored, context-sensitive interventions to support both breastfeeding and smoking cessation among postpartum women.

The barriers to breastfeeding are multifaceted and include historical injustices such as forced removal of AI/AN children into boarding schools [26] and Black enslaved women forced to breastfeed their master’s children [27]. Contemporary barriers include cultural stigma around breastfeeding [28], limited structural support in the workplace and lack of paid parental leave [29], disproportionality impacting women of color [30]. Additional hospital practices such as providing formula to low-income mothers without medical indication [31] may further undermine breastfeeding. Furthermore, formula companies frequently disseminate misleading information that overstates the benefits of their products compared to breastmilk [32, 33]). These systemic challenges compound the effects of postpartum smoking, particularly for marginalized communities.

Prior literature suggests a bidirectional relationship between breastfeeding and smoking behaviors, with longer breastfeeding duration associated with decreased postpartum smoking relapse [34, 35]. Although multiple postpartum smoking relapse interventions have been developed, maintaining long-term smoking abstinence following pregnancy remains challenging [16, 19, 36–40]. Providers should offer harm-reduction counseling, including strategies such as smoking after breastfeeding, avoiding smoking near the baby, and reducing cigarette consumption [40].

The strengths of this study include the use of a large, nationally representative dataset and the inclusion of underrepresented groups, particularly AI/AN women, enabling robust race-stratified analyses. Limitations include the reliance on self-reported use of cigarettes in the 2–6 months post-delivery may introduce recall bias or underreporting of tobacco use. Although the term “postpartum smoking” was used to quantify the use of cigarettes in the prior three months, given that some mothers took the survey two months postpartum they could have been referring to smoking while pregnant. There are likely unmeasured confounders, such as familial and community support, that could influence breastfeeding outcomes. WIC participation was included as an available proxy measure of socioeconomic disadvantage within PRAMS; however, WIC enrollment may not fully capture socioeconomic status, as many eligible families may not participate in the program. Additionally, immigration status and acculturation are not accounted for in the PRAMS data, which could potentially confound the findings, especially among Hispanic women who experience an increase in smoking prevalence and a decrease in breastfeeding rates with increased acculturation [41, 42]. PRAMS does not capture data regarding the use of smoking cessation strategies or intentions of quitting smoking. This study did not include other forms of nicotine. Although some information on e-cigarette use was available in PRAMS, the present analyses focused on cigarette smoking to maintain consistency in exposure definitions across study years and racial/ethnic subgroup analyses. Future studies should examine whether breastfeeding outcomes differ across tobacco product types. Finally, given the cross-sectional design, it cannot infer causality or determine the directionality of the association between postpartum smoking and breastfeeding outcomes. Future research should account for differences in social experiences across race and ethnic groups instead of using the race and ethnicity as a proxy.

This study of a race-stratified analysis of 2016–2022 PRAMS data expands existing knowledge on postpartum smoking and breastfeeding outcomes and reveals that racial disparities persist despite overall trends in smoking cessation. Addressing these disparities requires culturally tailored interventions that integrate smoking cessation support with breastfeeding promotion. While quitting smoking is ideal, healthcare providers should encourage mothers who smoke to continue breastfeeding while using harm-reduction strategies. Smoking cessation programs that are culturally tailored and integrating breastfeeding education into tobacco cessation programs are needed to promote harm-reduction strategies for postpartum smokers and improve both maternal and infant health outcomes.

## Data Availability

No datasets were generated or analysed during the current study.

## Code availability (software application or custom code)

All analyses were performed using Stata version 18 (StataCorp, College Station, TX). Custom Stata .do files used for data management and analysis are available from the corresponding author upon reasonable request.
